# Predictability of uncontrollable multifocal seizures – towards new treatment options

**DOI:** 10.1038/srep24584

**Published:** 2016-04-19

**Authors:** Klaus Lehnertz, Henning Dickten, Stephan Porz, Christoph Helmstaedter, Christian E. Elger

**Affiliations:** 1 Department of Epileptology, University of Bonn, Sigmund-Freud-Str. 25, 53105 Bonn, Germany; 2 Helmholtz-Institute for Radiation and Nuclear Physics, University of Bonn, Nussallee 14-16, 53115 Bonn, Germany; 3 Interdisciplinary Center for Complex Systems, University of Bonn, Brühler Straβe 7, 53175 Bonn, Germany

## Abstract

Drug-resistant, multifocal, non-resectable epilepsies are among the most difficult epileptic disorders to manage. An approach to control previously uncontrollable seizures in epilepsy patients would consist of identifying seizure precursors in critical brain areas combined with delivering a counteracting influence to prevent seizure generation. Predictability of seizures with acceptable levels of sensitivity and specificity, even in an ambulatory setting, has been repeatedly shown, however, in patients with a single seizure focus only. We did a study to assess feasibility of state-of-the-art, electroencephalogram-based seizure-prediction techniques in patients with uncontrollable multifocal seizures. We obtained significant predictive information about upcoming seizures in more than two thirds of patients. Unexpectedly, the emergence of seizure precursors was confined to non-affected brain areas. Our findings clearly indicate that epileptic networks, spanning lobes and hemispheres, underlie generation of seizures. Our proof-of-concept study is an important milestone towards new therapeutic strategies based on seizure-prediction techniques for clinical practice.

In terms of both prevalence and cumulative incidence, epilepsy is one of the most common serious neurological disorders, affecting approximately 65 million people worldwide^1^. In Europe alone, the costs attributable to active epilepsy had been estimated to exceed € 20 billion per year^2^,^3^,^4^. Despite modern drug therapies^5^ and advanced surgical methods^6^, around 20–30% of patients remain poorly treated or untreated, requiring comprehensive care to address the adverse events of medical treatment, quality of life issues, and comorbid disorders^7^,^8^.

A large number of patients with chronic intractable epilepsy present seizure onsets and/or structural abnormalities that are either too diffuse or in a sensitive area of eloquent cortex or bilateral (multifocal). These patients are usually not amenable to traditional EEG-guided resections^9^, and surgical treatment of brain areas, even if abnormal, invariably leads to new deficits. An alternative treatment option for controlling previously uncontrollable seizures would consist of identifying a transitional pre-seizure state in critical areas of the brain^10^,^11^,^12^,^13^,^14^,^15^ combined with a counteracting influence^16^,^17^ to prevent ictogenesis. Such a method has a high potential for preventing injuries, and allowing subjects with uncontrollable multifocal or poorly localized seizures to re-enter society. Previous research on the identification of a transitional pre-seizure state, however, primarily employed data from patients with clearly circumscribed seizure onsets and post-operative complete seizure control. To further advance this field, particularly with respect to the future clinical use of seizure-prediction and -prevention systems, here we report the results of our study to assess the feasibility of state-of-the-art, electroencephalogram-based seizure-prediction techniques in patients with chronic intractable epilepsy.

## Results

**Figure 1:**
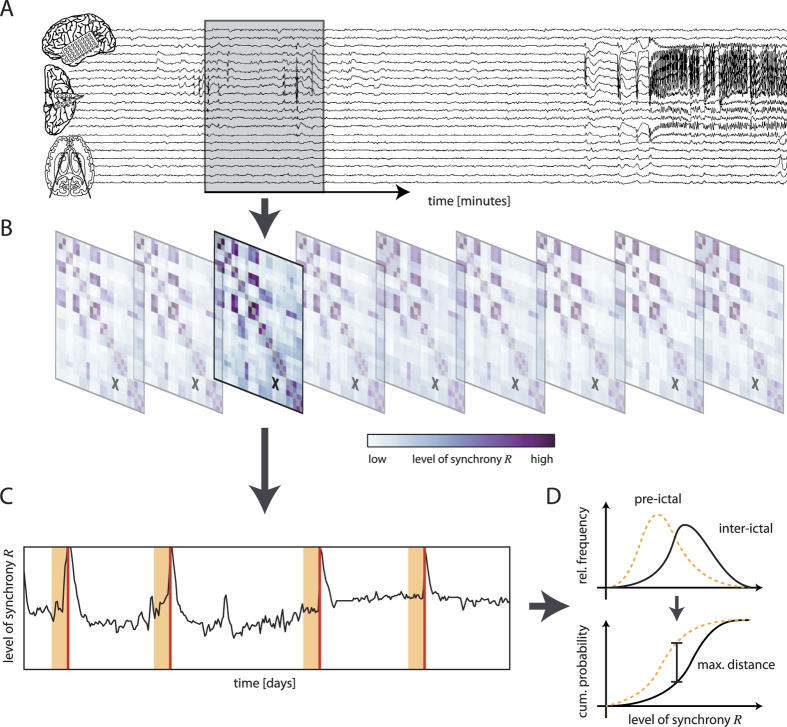
Identifying critical brain areas I. (**A**) The level of synchrony *R*^25^ between iEEG data from all pairs of sampled brain areas is estimated in a sliding-window fashion, resulting in sequence of symmetric synchrony matrices (**B**). (**C**) Temporal sequence of the level of synchrony between a pair of brain areas (marked with X in (**B**)). Seizures are marked as red vertical lines, and their assumed pre-ictal periods are indicated as yellow-shaded areas. (**D**) Corresponding frequency distributions (top) and cumulative distribution functions (bottom) of the level of synchrony for inter-ictal (black, solid line) and pre-ictal data (yellow, dashed line). Separability of inter-ictal and pre-ictal distributions is indicated by the maximum distance between cumulative distribution functions and is used for an automated pre-selection of pairs of brain areas.

**Figure 2:**
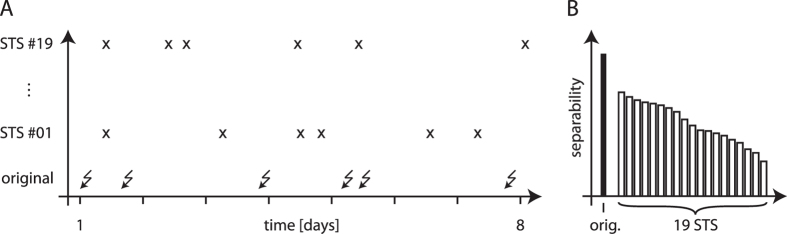
Identifying critical brain areas II. Evaluating predictive performance with seizure time surrogates (STS)^46^. (**A**) Exemplary sequence of original seizure times (lower trace, bolts) and surrogate times (upper traces, crosses). The latter were derived from a random permutation of the original inter-seizure intervals and the interval from the first seizure back to an arbitrarily defined starting point. (**B**) Predictive performance is considered significant (*p* < 0.05) if separability of inter-ictal and pre-ictal distributions for original seizure times exceeds the maximum one obtained with 19 STS. Note that the available data limits the number of STS confirming with the requirement for independence^47^, and hence the minimum error probability which prevents correcting for multiple testing at this stage of analysis.

**Figure 3:**
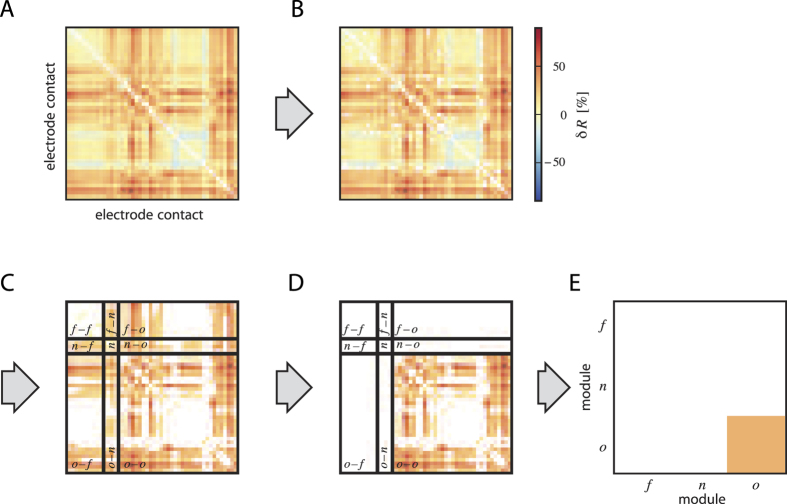
Identifying critical brain areas III. Example of singling out pairs of electrode contacts whose time-dependent changes in synchrony may carry potential information about pre-seizure states via downstream statistical analysis. (**A**) Symmetric matrix of relative change in synchrony 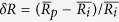 for each pair of electrode contacts (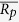 and 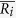 denote the medians of *R*-values from the pre-ictal and inter-ictal periods). (**B–D**) Matrices after steps 1–3 of analyses (see text). This procedure allowed a reduction of pairs of electrode contacts by a factor of 23 in group 1 and by a factor of 20 in group 2. (**E**) Symmetric matrix of predictive interactions between functional modules (focal (*f*), neighborhood (*n*), other (*o*)): each color-coded entry represents the mean relative change in synchrony obtained from averaging over all *δR*-values from the respective critical module combinations (**D**).

A large number of analysis techniques and prediction algorithms as well as statistical approaches to evaluate prediction performance have been proposed to identify a transitional pre-seizure state and the brain region(s) from which seizure precursors emerge^12^,^14^. However, the rather restricted knowledge about seizure precursor dynamics in patients with chronic intractable epilepsy renders an a priori choice of methods and algorithms quite difficult. We therefore designed a retrospective feasibility study to provide proof-of-concept data for seizure predictability in patients with uncontrollable multifocal, drug-resistant epilepsy. We probed for the existence of seizure precursors in continuous, multi-contact, multi-day, intracranial electroencephalographic (iEEG) recordings capturing more than 200 seizures from two patient groups (see Methods and Materials). Group 1 comprised patients for which multiple, non-resectable seizure onset zones (SOZs) had been identified using established presurgical evaluation techniques^18^. Patients from group 2 achieved complete seizure control after resection of a single SOZ, and these patients were included for the purpose of comparison. We employed a widely used seizure-prediction technique, for which a prediction performance clearly exceeding the chance level (as evidenced by robust statistical validation) has repeatedly been shown^19^,^20^,^21^,^22^,^23^,^24^. In addition, we made use of several sophisticated statistical techniques that helped us to avoid drawing incorrect conclusions about seizure precursors and about critical brain areas, which may arise due to various influencing factors and due to issues of multiple comparisons. Our analysis strategy is summarized in Figs 1, 2, 3.

First, we assessed—in a frequency-adaptive and time-resolved manner—the level of synchrony *R*^25^ between each pair of sampled brain areas from their iEEG data (see section Methods and Materials and Fig. 1A–C). Next we used a commonly employed statistical approach^12^ to compare the distributions of *R* values from the inter-ictal with those from an assumed pre-ictal period^19^,^20^,^22^. Given that seizure generation is likely to take place over minutes to hours^25^,^26^,^27^,^28^, with multiple mechanisms occurring over different stages in ictogenesis, and in accordance with typical prediction times for synchrony-based measures described in the literature^19^,^22^,^25^,^29^,^30^,^31^, we assumed the pre-ictal period to last for T_pre_ = 4 h. In order to not bias our analyses with effects from the seizure and particularly from the post-ictal period^32^,^33^, we discarded data from the 30 min interval after the onset of a seizure (in cases where the time between two successive seizures was less than T_pre_ − 30 min, the maximum amount of data available, i.e., from seizure onset back to the end of the post-ictal phase of the preceding seizure, was used instead, see Tables 1 and 2).

We then identified critical brain areas that are likely associated with the emergence of seizure precursors, by singling out pairs of electrode contacts whose time-dependent changes in synchrony may carry potential information about pre-seizure states. To do so, we performed the following steps of analysis:

1. We collected all pairs of electrode contacts that had a significant ability to separate inter-ictal from pre-ictal epochs (i.e., their prediction performances; see Fig. 1D; Kolmogorov-Smirnov test; *p* < 0.05 after Bonferroni correction);
2. We tested whether the prediction performance of a given pair is better than random by testing it against the null hypothesis of the non-existence of a pre-seizure state: For this purpose, we employed the concept of seizure time surrogates (see Fig. 2) that also allowed us to account for possible confounding influences such as seizure clustering, daily rhythms, and changes in anticonvulsive medication. We denote pairs with a prediction performance better than random (*p* < 0.05) as *critical electrode pairs* (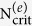 in Tables 1 and 2);
3. We registered to which combination of functional modules—focal (*f*), neighborhood (*n*), and other (*o*)— critical electrode pairs belong to (i.e., *f*–*f*, *n*–*n*, *o*–*o*, *f*–*n*, *f*–*o*, or *n*–*o*). Modules are described in detail in Methods and Materials. We then checked for each patient, whether some module combination preferentially contained critical electrode pairs, given the varying number of electrode contacts within each module (hypergeometric test; *p* < 0.05). We denote these as *critical module combinations*.

This entire procedure, exemplarily illustrated in Fig. 3, allowed us to reduce the initial number of pairs of sampled brain areas (on average, 2714 in group 1 and 1243 in group 2) to a smaller number of critical electrode pairs in critical module combinations (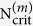 in Tables 1 and 2; on average, 118 in group 1 and 63 in group 2) that represent critical areas of the brain carrying potential information about transitional pre-seizure states.

**Figure 4:**
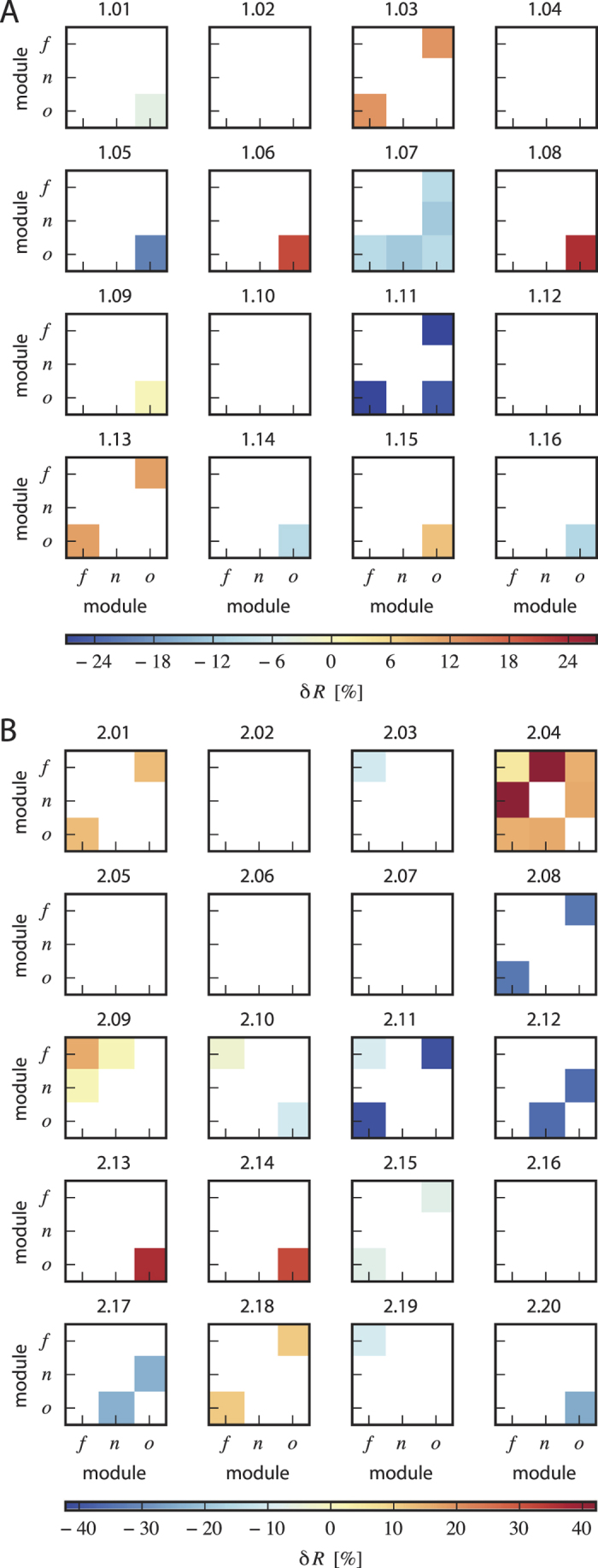
Critical module combinations (carrying predictive information) for each patient. Symmetric matrix of predictive interactions between functional modules (focal (*f*), neighborhood (*n*), other (*o*)): each color-coded entry represents the mean relative change in synchrony obtained from averaging over all *δR*-values from the respective critical module combinations identified in each patient (see Fig. 3E). (**A**) patients with chronic intractable epilepsy (group 1; patient-codes as in Table 1); (**B**) control group (group 2; patient-codes as in Table 2).

Figure 4 summarizes our main findings for each patient. Among the sixteen patients with chronic intractable epilepsy (group 1), we observed ten patients with predictive indications from interactions between brain regions not related to initial ictal discharges (*o*–*o* interactions). Four patients presented with predictive *f*–*o* and one patient with predictive *n*–*o* interaction (note that a patient may contribute to more than one critical module combination). Among the twenty patients in the control group (group 2), we observed six patients each with predictive indications from interactions within the SOZ (*f*–*f* interactions) as well as from interactions between the SOZ and remote brain regions (*f*–*o* interactions). Four patients presented with predictive *o*–*o* interactions, three with predictive *n*–*o* and two with predictive *f*–*n* interactions.

**Figure 5:**
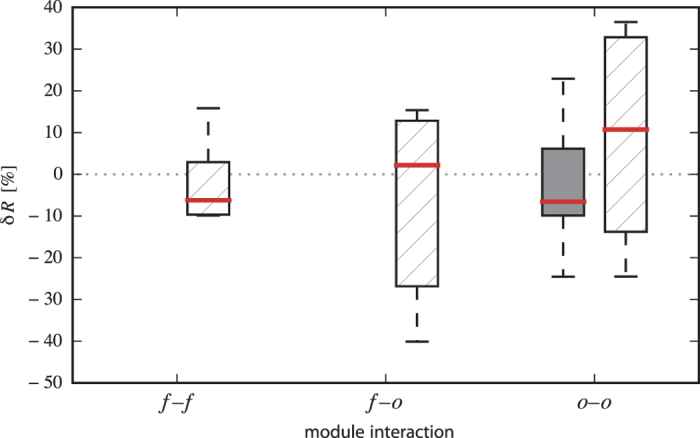
Comparison of the distributions of pre-seizure changes in synchrony in critical brain areas between patient groups. Boxplots of the average relative change in synchrony 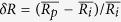 between critical brain areas (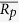 and 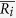 denote the medians of *R*-values from the respective pre-ictal and inter-ictal periods) for patients with chronic intractable epilepsy (group 1, filled box) and for patients in the control group (group 2, hatched boxes). Module interactions: *f*–*f* (within the seizure onset zone), *f*–*o* (between seizure onset zone and remote brain areas), and *o*–*o* (between remote brain areas). Only data from the final stage of statistical analyses are shown. Bottom and top of a box are the first and third quartiles, and the (red) band inside a box is the median. The ends of the whiskers represent the minimum and maximum of the data.

We checked whether the aforementioned module combinations were preferentially critical across a patient group. For the critical module combinations in group 1, we found that only the number of patients with interactions between brain regions not related to initial ictal discharges (*o*–*o* interactions) exceeded what was expected by chance (hypergeometric test; *p* < 0.05). In these non-affected brain regions, a loss of synchrony (−6.6% on average) can be regarded as a potential seizure precursor (see Fig. 5). For the critical module combinations in the control group (group 2), the number of patients with alterations in synchrony of within-module interactions of the SOZ (*f*–*f*) and of remote brain areas (*o*–*o*) as well as their between-module interactions (*f*–*o*) exceeded what was expected by chance (hypergeometric test; *p* < 0.05). Potential seizure precursor were characterized by a slightly decreased pre-ictal synchrony (−6.2% on average) within the SOZ but also by a slightly increased pre-ictal synchrony between SOZ and other brain regions (+2.2% on average) and a more pronounced increase of synchrony (+10.7% on average) between remote brain regions (see Fig. 5).

In both patient groups, the interaction dynamics of brain areas clearly beyond the seizure onset zone turned out to carry predictive information about upcoming seizures. Interestingly, these interactions covered virtually all spatial scales, ranging from short-range (neighboring electrode contacts) via medium-range (within lobes) to long-range interactions (across lobes and even hemispheres). Potential seizure precursors could, however, be observed much more often for medium-range and long-range interactions (data not shown).

## Discussion

Our retrospective study is the first to demonstrate feasibility of seizure prediction in patients with multifocal epilepsies. These are among the most difficult epileptic disorders to manage since they are often refractory to medical therapy and not treatable by resective epilepsy surgery. Our findings demonstrate brain areas clearly beyond the seizure onset zone(s) to carry predictive information about upcoming seizures. This is in line with many previous reports on the high relevance of brain outside of the seizure onset zone in focal epilepsies^29^,^34^,^35^,^36^,^37^,^38^, although their relevance may be debated due to limitations arising from applying various ad hoc preselection criteria or from statistical issues^22^. Our findings are nonetheless unexpected, since they point to different seizure-generating mechanisms underlying focal and multifocal seizures. Not only are the brain regions involved different (focal seizures: onset zone and remote, non-affected brain regions; multifocal seizures: remote, non-affected regions only), but also the nature of changes in pre-ictal changes in synchrony appears to be different (focal seizures: pre-ictal increase and decrease, depending on involved brain regions; multifocal seizures: pre-ictal decrease only). Our findings thus challenge the traditional view of autonomous brain regions underlying ictogenesis in multifocal epilepsies and call for follow-up studies to further our understanding of seizure generation in these epilepsies. Our findings strongly emphasize large-scale epileptic networks—spanning affected and non-affected lobes and hemispheres—to underlie ictogenesis, thus providing novel insights into pathways regulating seizure generation and that may be dysfunctional in these disorders. Knowledge about these network constituents may be refined with methods from network theory^39^ and may elucidate targets for systemic approaches that strive for individualized therapeutic interventions^40^,^41^,^42^ aiming at preventing seizure generation in multifocal epilepsies.

Our study had some limitations. It was based on data recorded during the presurgical evaluation, and a number of variables (such as transient effects of surgery, medication tapering, sleep deprivation, etc.) could confound the identification of a transitional pre-seizure state. We therefore chose to not report on characteristics of prediction performance (such as sensitivity, specificity, prediction times, or the portion of time under false warning^12^). Future prospective studies based on electroencephalographic monitoring (or of other suitable modalities) in ambulatory patients together with specifically designed estimators for predictive performance^12^ will elucidate the clinical usefulness of the method.

With our study design, we obtained significant predictive information about upcoming seizures in about two thirds of patients with multifocal epilepsies. A similar ratio has been reported on earlier for EEG-based prediction studies in focal epilepsies^43^ as well as for surveys on epileptic prodromes (or premonitory symptoms)^44^. Notwithstanding limitations of prediction algorithms as well as conceptual and statistical issues related to surveys, this ratio might imply that seizure prediction may not be possible in every patient. Future studies might help answering the question whether patients with specific type of epilepsy but predictable seizures represent another phenotype than patients with non-predictable seizures.

Our feasibility study shows that pre-seizure states can be identified in patients with multifocal epilepsies. This could lead to new therapeutic strategies to efficiently control generation of previously uncontrollable seizures, e.g. via closed-loop, on-demand electrophysiological and/or behavioral brain stimulation. This approach could even be translated to neurological diseases not considered for such interventions so far.

## Methods

### Study Design and Participants

We did a retrospective feasibility study to provide proof-of-concept data for seizure predictability in patients with uncontrollable multifocal epilepsy. Between 2002 and 2012, 380 patients with drug-resistant epilepsy underwent presurgical evaluation with intracranial electroencephalographic recordings. From this sample, we identified 16 patients for which multiple, non-resectable seizure onset zones (SOZs) had been identified and for which at least 24 hours of recording were available that captured at least one seizure. We assigned these patients (10 women, mean age: 32 years, range 15–62 years; mean age at onset of epilepsy: 13 years, range 0–35 years; mean duration of epilepsy: 19 years, range 4–49 years, see Table 1) to group 1. From the same sample, we randomly selected 20 patients for which complete post-operative seizure control could be achieved after resection of a single SOZ and for which at least 24 hours of recording were available that captured at least one seizure. We assigned these patients (8 women, mean age: 34 years, range 15–64 years; mean age at onset of epilepsy: 13 years, range 0–35 years; mean duration of epilepsy: 22 years, range 2–53 years, see Table 2) to group 2. All patients had signed informed consent that their clinical data might be used and published for research purposes. The study protocol had previously been approved by the ethics committee of the University of Bonn, and methods were carried out in accordance with the approved guidelines.

Patients received different antiepileptic drugs (AEDs) with different mechanisms of action, and the majority of patients were under combination therapy with two or more AEDs. During presurgical evaluation AEDs were reduced in a patient-specific manner, and many patients did not have discontinuation of all AEDs.

### Intracranial EEG recordings

We recorded intracranial electroencephalograms (iEEG) from chronically implanted intrahippocampal depth electrodes and subdural grid- and/or strip-electrodes (cf. Fig. 1A). Depth electrodes were equipped with 10 cylindrical contacts of length 2.5 mm and an intercontact distance of 4 mm. Strip electrodes consisted of 4 or 8 contacts with an intercontact distance of 10 mm. Grid electrodes had 8 × 4 or 8 × 8 contacts with an intercontact distance of 10 mm. Decisions regarding placement of electrodes were purely clinically driven and were made independently of this study. Data were band-pass-filtered between 1–45 Hz, sampled at 200 Hz (sampling interval 5 ms) using a 16 bit analog-to-digital converter, and referenced against the average of two electrode contacts outside the presumed focal region. Reference contacts were chosen individually for each patient. For the patients in group 1, the recording with, on average, 66 contacts lasted 77 days during which 100 clinical seizures (6 seizures/patient, range 1–25, see Table 1) occurred. For the patients in group 2, the recording with, on average, 45 contacts lasted 98 days during which 108 clinical seizures (5 seizures/patient, range 1–24, see Table 2) occurred. The time of seizure onset was visually identified on the iEEG as the time of earliest clear change from the patient’s baseline or normal background iEEG that eventually led to an electrographic seizure. Subclinical seizures were neglected in our analyses.

### Functional modules

Since number and anatomical locations of intracranial electrodes were adapted to the patients’ needs and were thus highly non-uniform, we assigned electrode contacts to functional modules in order to facilitate within- and between-group comparisons:

- Module *f* (*focal*): contacts where first ictal discharges were recorded^18^ (on average 38.5% (range 3.4–90.0%) of contacts in group 1 and 15.2% (range 1.9–27.8%) of contacts in group 2),
- Module *n* (*neighborhood*): contacts not more than two contacts distant to those from *f* (on average 1.6% (range 0.0–7.5%) of contacts in group 1 and 9.2% (range 0.0–22.2%) of contacts in group 2),
- Module *o* (*other*): all remaining contacts (on average 59.9% (range 10.0–94.3%) of contacts in group 1 and 75.6% (range 50.0–91.0%) of contacts in group 2).

### Measuring the level of synchrony between brain areas

We calculated in a time-resolved manner (non-overlapping windows of 20.48 s duration; 4096 data points) the mean phase coherence *R*^25^ between phase time series that we derived by Hilbert-transforming iEEG recordings. *R* takes on values between 0 and 1, indicating either complete asynchrony or complete synchrony. Analyses were performed for every possible combination of pairs of electrode contacts (see Fig. 1A–C).

### Statistical analysis

We used the (two-sample) Kolmogorov-Smirnov statistic^45^ to quantify the distance between the cumulative distribution functions of inter-ictal and pre-ictal levels of synchrony (derived from the pooling of all pre-ictal and inter-ictal *R* values). For downstream statistical analyses, we only considered distance estimates for which we attained a significance level of *p* < 0.05 after Bonferroni correction.

We performed hypergeometric tests to check whether our approach preferentially assigned critical electrode pairs to module combinations due the strongly varying number of electrode contacts *N*_*c*_ within each functional module. If the hypergeometric *p*-value (calculated as the probability of randomly drawing a given number *k* or more critical pairs of brain areas from the population in *N*_*c*_ total draws) was less than 0.05, we considered this number significant.

We also performed hypergeometric tests to check whether a given module combination was preferentially critical across a patient group. If the hypergeometric *p*-value (calculated as the probability of randomly drawing a given number *k* or more patients for which a given module combination was critical, given the total number of critical module combinations) was less than 0.05, we considered the respective number of patients significant.

## Additional Information

**How to cite this article**: Lehnertz, K. *et al*. Predictability of uncontrollable multifocal seizures – towards new treatment options. *Sci. Rep.* **6**, 24584; doi: 10.1038/srep24584 (2016).

## Figures and Tables

**Table 1:** Demographics of patients form group 1 (age and duration of epilepsy (D_e_) in years; w.p.f., without pathological findings; AHS, Ammon’s horn sclerosis; bilat., bilateral; N_szr_, number of clinical seizures; total (D_i_, D_p_) and average (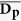) duration of inter-ictal and pre-ictal periods in hours; 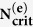, total number of critical electrode pairs; 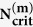, number of critical electrode pairs in critical module combinations).

	**Age**	**Sex**	**D**_**e**_	**MRI**	**N**_**szr**_	**D**_**i**_	**D**_**p**_	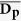	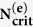	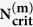
Patient 1.01	54	Male	45	w.p.f.	1	274.1	4.0	4.0	223	220
Patient 1.02	38	Male	4	bilat. AHS	9	17.9	20.8	2.3	67	0
Patient 1.03	15	Female	10	bilat. AHS	11	145.8	17.8	1.6	161	91
Patient 1.04	34	Female	15	bilat. AHS	9	82.3	13.4	1.5	0	0
Patient 1.05	24	Female	22	bilat. AHS	6	28.6	13.5	2.3	62	28
Patient 1.06	29	Male	16	bilat. AHS	10	97.4	23.3	2.3	159	125
Patient 1.07	17	Female	13	Dysplasia	1	35.1	4.0	4.0	190	117
Patient 1.08	45	Male	23	w.p.f.	4	123.9	9.1	2.3	477	292
Patient 1.09	26	Female	6	w.p.f.	7	156.1	23.3	3.3	52	44
Patient 1.10	62	Female	49	Dysplasia	3	95.9	9.7	3.2	125	0
Patient 1.11	29	Female	25	bilat. AHS	25	146.4	24.1	1.0	826	639
Patient 1.12	19	Male	9	bilat. AHS	2	81.4	7.5	3.8	125	0
Patient 1.13	26	Female	18	w.p.f.	3	127.6	11.7	3.9	10	9
Patient 1.14	26	Male	25	bilat. AHS	2	26.1	7.3	3.7	127	107
Patient 1.15	29	Female	12	w.p.f.	5	29.7	9.0	1.8	94	86
Patient 1.16	40	Female	13	w.p.f.	2	141.1	8.0	4.0	153	107
∅	32		19		6	100.4	12.9	2.8	182	118

**Table 2:** Demographics of patients form group 2 (age and duration of epilepsy (D_e_) in years; w.p.f., without pathological findings; AHS, Ammon’s horn sclerosis; FCD, focal cortical dysplasia; N_szr_, number of clinical seizures; total (D_i_, D_p_) and average (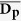) duration of inter-ictal and pre-ictal periods in hours; 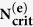, total number of critical electrode pairs; 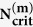, number of critical electrode pairs in critical module combinations).

	Age	Sex	D_e_	MRI	N_szr_	D_i_	D_p_	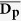	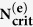	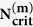
Patient 2.01	34	Male	29	Dysplasia	11	87.9	32.3	2.9	82	27
Patient 2.02	64	Female	53	Cavernoma	1	26.0	4.0	4.0	0	0
Patient 2.03	25	Female	20	w.p.f.	4	169.7	10.0	2.5	3	3
Patient 2.04	22	Male	23	w.p.f.	12	97.4	25.3	2.1	856	353
Patient 2.05	57	Male	50	Hamartia	5	74.1	13.9	2.8	49	0
Patient 2.06	25	Male	24	AHS	4	33.7	11.3	2.8	146	0
Patient 2.07	38	Male	15	AHS	4	70.7	12.8	3.2	8	0
Patient 2.08	44	Female	30	FCD	4	105.3	10.3	2.6	20	14
Patient 2.09	52	Male	51	AHS	2	129.3	8.0	4.0	21	6
Patient 2.10	25	Male	13	FCD	12	24.7	18.6	1.5	86	56
Patient 2.11	26	Female	10	FCD	1	124.9	4.0	4.0	41	26
Patient 2.12	54	Female	48	FCD	2	116.5	4.0	2.0	272	83
Patient 2.13	27	Female	15	w.p.f.	2	159.0	8.0	4.0	522	421
Patient 2.14	37	Male	5	AHS	4	94.9	11.8	3.0	496	262
Patient 2.15	37	Male	2	w.p.f.	7	75.6	21.7	3.1	71	23
Patient 2.16	35	Male	6	w.p.f.	1	20.5	3.7	3.7	0	0
Patient 2.17	15	Female	11	FCD	3	36.1	8.0	2.7	10	7
Patient 2.18	24	Male	4	w.p.f.	2	102.7	8.0	4.0	362	80
Patient 2.19	22	Male	17	Lesion	3	24.3	11.7	3.9	4	2
Patient 2.20	27	Female	13	FCD	24	59.3	17.5	0.7	38	20
∅	34		22		5	81.6	12.2	3.0	136	63
